# Diabetes-Associated Susceptibility to Tuberculosis: Contribution of Hyperglycemia vs. Dyslipidemia

**DOI:** 10.3390/microorganisms9112282

**Published:** 2021-11-02

**Authors:** Minh Dao Ngo, Stacey Bartlett, Katharina Ronacher

**Affiliations:** 1 Mater Research Institute, The University of Queensland, Translational Research Institute, Brisbane, QLD 4102, Australia; minhdao.ngo@uq.net.au (M.D.N.); stacey.bartlett@mater.uq.edu.au (S.B.); 2 Australian Infectious Diseases Research Centre, The University of Queensland, Brisbane, QLD 4072, Australia

**Keywords:** tuberculosis, *Mycobacterium tuberculosis*, diabetes, hyperglycemia, dyslipidemia, cholesterol, triglycerides

## Abstract

Diabetes is a major risk factor for tuberculosis (TB). Diabetes increases the risk of the progression from latent tuberculosis infection (LTBI) to active pulmonary TB and TB patients with diabetes are at greater risk of more severe disease and adverse TB treatment outcomes compared to TB patients without co-morbidities. Diabetes is a complex disease, characterised not only by hyperglycemia but also by various forms of dyslipidemia. However, the relative contribution of these underlying metabolic factors to increased susceptibility to TB are poorly understood. This review summarises our current knowledge on the epidemiology and clinical manifestation of TB and diabetes comorbidity. We subsequently dissect the relative contributions of body mass index, hyperglycemia, elevated cholesterol and triglycerides on TB disease severity and treatment outcomes. Lastly, we discuss the impact of selected glucose and cholesterol-lowering treatments frequently used in the management of diabetes on TB treatment outcomes.

## 1. Introduction

Approximately 2 billion people worldwide are estimated to be infected with *Mycobacterium tuberculosis* (Mtb) [1]. Of those, 5–10% will develop active tuberculosis (TB) in their lifetime. In 2019, 10 million people suffered from active TB, which caused 1.5 million deaths [2]. The cost of TB prevention, diagnosis and treatment is expected to double from 6.5 billion USD in 2020 to 13 billion USD per year by 2022. This highlights that TB remains a global health threat and a significant financial burden. The proposed 2020 milestone of a 20% reduction in TB cases and a 35% reduction in deaths between 2015 and 2020 was not reached, although global TB incidence rates and annual death numbers are decreasing [2]. The goal of achieving an 80% decrease in TB incidence or a 90% decrease in annual death by 2030 will not be met without intensified effort. Several factors including malnutrition, diabetes, smoking and alcohol abuse contribute to an increase in the susceptibility to TB. With the rapid rise in type 2 diabetes (T2D) prevalence in developing countries, a significant risk factor for TB is emerging. T2D affects one in 11 individuals globally (463 million people), and almost 50% of T2D patients are undiagnosed [3]. It is projected that the number of T2D patients will increase by 51% reaching 700 million people in 2045 [3], with most cases living in developing countries, where TB is endemic [4]. Hence, an increase in the global burden of T2D-TB comorbidity is expected in the next decade.

The COVID-19 pandemic has adversely affected the global efforts to control both TB and T2D, especially in low- and middle-income countries. According to a WHO statement made based on surveys from 163 countries, 49% of the surveyed countries have seen a reduction in access to treatments for T2D [5]. Globally, if the number of new TB cases detected over a 3-month period during the pandemic is reduced by 25% compared to the level of detection prior to the pandemic, an additional 190,000 TB deaths are estimated [6]. It is inevitable that the pressures placed on healthcare systems by the COVID-19 pandemic around the world will likely result in a delay in the fight against both TB and T2D.

This review article discusses the current knowledge on the association between TB and T2D with a particular focus on the contribution of metabolic factors, including body mass index (BMI), hyperglycemia and dyslipidemias to increased susceptibility to TB.

## 2. Impact of T2D on Latent TB Infection and Active TB Disease

### 2.1. T2D Increases the Risk of Latent TB Infection

Latent TB infection (LTBI), defined as asymptomatic infection with Mtb, usually precedes active pulmonary TB. Studies assessing the association between T2D and LTBI are most valuable if conducted in low-TB-burden countries, as baseline LTBI positivity is high irrespective of T2D status in high-TB-burden countries such as South Africa [7]. In a cross-sectional study conducted in a health clinic in Atlanta, GA, USA, LTBI was significantly higher amongst patients with T2D (43.4%) and pre-diabetes (39.1%) compared to those without T2D (25.9%). Furthermore, a strong association between T2D and LTBI ([aOR] 2.3, 95% CI 1.2–4.5) was demonstrated in this cohort of patients [8]. This relationship between TB and LTBI was also identified by Lee et al., who found T2D to be associated with a small but significantly increased risk of LTBI (pooled OR of 1.18, 95%CI 1.06–1.30) [9]. In a large-cross sectional analysis using US National data, Barron et al. reported a significantly higher association of T2D and LTBI (aOR 1.90, 95%CI 1.15–3.14) compared to adults without T2D [10]. In contrast, a hospital-based study in Atlanta found that the LTBI prevalence was higher in patients without T2D (14.7%, 5/34) compared to patients with newly diagnosed T2D (9.2%, 9/98) [11]; however, these results are based on small sample sizes and could be confounded by other underlying health conditions requiring hospital admission. Altogether, these studies provide evidence for an increased risk of primary infection with Mtb in patients with T2D compared to otherwise healthy individuals.

### 2.2. T2D Increases the Risk of Active TB

If the host immune response is unable to contain the Mtb infection, the progression from asymptomatic LTBI to active TB disease can occur. The association between T2D and active TB is well established and has been reviewed elsewhere [12,13,14]. Accumulating data from 44 studies, including prospective, retrospective and case control studies, Al Rifai and colleagues showed that T2D patients had a 2–4-fold increased risk of active TB [15]. Cohort studies from countries with a low and moderate incidence of TB incidence demonstrated a more than 2-fold increased TB risk among T2D patients with pooled relative risk (RR) (2.03, 95%CI 1.62–2.55) [9]. The latest WHO report estimates that approximately 350,000 TB cases were attributable to T2D in 2019 [2]. There is now agreement that T2D predisposes individuals to developing active TB and that its contribution to global TB prevalence will increase further with the rise of T2D prevalence. Consequently, several clinical trials for TB preventative therapy in LTBI-positive T2D patients have been initiated or are currently ongoing [16,17].

### 2.3. T2D Increases the Risk of Multidrug-Resistant TB

Resistance to antibiotic TB therapy is rising globally with close to half a million TB cases resistant to one of the most potent TB drugs, rifampicin [2]. Of those, 78% had multidrug-resistant TB (MDR-TB). The treatment of MDR-TB takes up to 20 months, with a global treatment success rate of only 57% [2]. Extensively drug-resistant TB (XDR TB) (resistance to rifampicin, isoniazid, any fluoroquinolone and at least one the following: levofloxacin, moxifloxacin, bedaqualine and linezolid) was identified in close to 13,000 cases in 2019 [2].

A growing body of literature has linked T2D with an increased risk of MDR-TB. In a meta-analysis including 24 observational studies, Tegene et al. revealed that T2D is associated with higher rates of MDR-TB (OR = 1.97, 95% CI = 1.58–2.45), irrespective of country income level [18]. Similarly, Huangfu and colleagues found that T2D was associated with a two-fold increased risk of MDR-TB among TB patients (OR 1.98, 95%CI 1.51–2.60) based on robust evidence from 104 publications [19].

Using whole-genome sequencing to examine drug resistance mutations in Mtb isolates of TB patients with T2D, Ruesen et al. demonstrated that in a cohort in Peru T2D was associated with significantly more mutations conferring resistance to isoniazid and ethionamide (Rv1482c-fabG1) and fluoroquinolone (gyrA), as well as a trend towards more mutations for rifampicin (rpoB) resistance [20]. The association between T2D and drug-resistant mutations was evident even among patients with newly diagnosed TB and was independent of the levels of glycemic control determined by HbA1c [20], thus suggesting that previous anti-TB treatment does not account for the higher risk of MDR-TB in TB-T2D patients.

Interestingly, lower concentrations of isoniazid and pyrazinamide were detected in serum from TB patients with T2D compared to TB patients without T2D [21,22]. These reduced systemic concentrations of antibiotics may contribute to the development of drug resistance. The reasons for lower antibiotic concentrations in T2D patients remain to be elucidated but are possibly linked to either the body weight distribution due to the generally higher BMI in T2D patients or more rapid metabolic breakdown of the TB treatment.

## 3. T2D Increases TB Disease Severity and the Risk of Adverse TB Treatment Outcomes

T2D patients who develop active TB frequently have more severe disease on chest X-ray, delayed culture conversion and higher sputum smear grades [23]. Higher smear grades in patients with TB-T2D co-morbidity are indicative of higher lung mycobacterial burdens, suggesting that TB patients with T2D are more infectious than TB patients without co-morbidities [23]. A recent study from Brazil confirmed that TB patients with any form of dysglycemia (T2D or pre-diabetes) are more likely to transmit Mtb in a household contact setting [24].

TB patients with T2D frequently have more severe disease upon chest X-ray at diagnosis with more cavities and parenchymal lesions [25,26]. Bilateral pulmonary involvement and extensive pulmonary disease was also found on CT scans in TB patients with underlying T2D [27,28]. Huang and colleagues showed that T2D patients with poor glycemic control (HbA1c > 8%) were more likely to present with atypical findings upon chest X-ray and thoracic CT scans, such as advanced extensive lesions (*p* < 0.001), more cavities (*p* < 0.001) and all-lobe involvement (*p* = 0.041) [28]. In contrast, one study found that T2D patients with TB presented with lower lung cavitary lesions compared to TB patients without T2D [29]. Differences in these findings could potentially result from differences in median age and levels of glycemic control in the respective study cohorts. In addition, dyslipidemias may contribute to the differential clinical manifestation of TB and have been shown to be highly variable in T2D patients across different ethnicities [7].

Several retrospective studies have demonstrated adverse TB treatment outcomes and higher mortality in TB patients with T2D [19,30]. A recent prospective study following more than 700 individuals from West India showed that T2D significantly increased the risk of early mortality during TB treatment (aHR, 4.36; 95% CI, 1.62–11.76) [31]. A multi-center prospective cohort study from Brazil demonstrated that participants with diabetes but not prediabetes are at higher risk of having an unfavourable outcome (1.76 and 2.45 times separately from two different cohorts) and an increased risk of death (1.93 and 2.16 times) [32].

A different study, also conducted in India, surprisingly found that poorly controlled T2D was not associated with higher odds of adverse TB treatment outcomes among TB patients with normal or high BMI and was associated with better TB outcomes among patients with low BMI [33]. These findings highlight that T2D is a complex disease and suggests that subtle metabolic sub-phenotypes (beyond the crude classification of T2D) may be more susceptible or resistant to adverse TB treatment outcomes.

Although T2D in Western populations is often associated with obesity, a significant proportion of T2D cases, particularly in Asian populations, do not have a high BMI [34]. A prospective cohort study of 225 new pulmonary TB patients with comorbid T2D in India found that low and normal BMI were more common among TB patients with T2D than high BMI (88% vs. 12%) [31]. Similar proportions between T2D with and without low BMI was also reported by Kubiak et al. in a cross-sectional analysis of active TB cases in southern India (90.3% vs. 9.7%) [35]. These observation of lower BMI in T2D patients with TB compared to T2D patients are likely confounded by TB-associated wasting. The prevalence of active TB was 12 times higher in obese diabetic adults compared to overweight-obese adults without T2D and 2.5 times higher in T2D vs. non-diabetic patients with normal weight and was not different among underweight adults [35]. Consistently with this observation, several other studies reported that obesity in the absence of hyperglycemia protects against TB [36,37,38] and individuals with high BMI are less likely to die during TB treatment. Similar observations were made in a murine model [39].

Whether diabetes increases the risk of active TB more profoundly in the overweight and obese population and less so in underweight and low-BMI subjects requires further evaluation but carries important implications in Asian populations, where T2D develops at a lower BMI compared to other ethnicities [34]. Nevertheless, the association between T2D and adverse TB treatment outcomes underscores the need for T2D screening among newly diagnosed TB patients. This will allow for the appropriate clinical management of newly diagnosed T2D and will improve TB treatment outcomes in patients living with T2D.

## 4. Contribution of Hyperglycemia to TB Disease Severity and Adverse TB Treatment Outcomes

### 4.1. T2D-Related Chronic Hyperglycemia

T2D is characterised by insulin resistance and the progressive loss of beta cell mass and/or function, which leads to chronic hyperglycemia [40]. The impact of the severity of hyperglycemia on the clinical manifestation of TB and TB treatment outcomes has been the focus of several studies. Using large primary care data from the UK, Critchley et al. showed that T2D patients with poor glycemic control (HbA1c > 11%) had an elevated risk for hospitalisation for various type of infections, including TB (incidence rate ratio: 4.70), irrespective of age [41]. Optimal control of blood glucose (HbA1c 6–7%) reduced risk of hospitalisation (IRR 1.41 vs. 4.70), but these well-controlled T2D patients were still at a higher risk compared to matched controls without T2D, suggesting that metabolic factors other than hyperglycemia contribute to increased susceptibility to infections in T2D [41]. Poor glucose control also increased the risk of mortality, as reported by Chiang et al. in a cohort study in Taiwan. The authors reported higher mortality among TB patients with Hba1c > 9% compared to those with HbA1c < 7% (6% vs. 18%) [42].

Furthermore, poor glucose control worsens the response to TB treatment. Only 47% of TB-T2D patients with an average HbA1c of 10.7% obtained sputum culture conversion by month 2 of TB treatment, compared to sputum culture conversion rates of 73% in TB patients without T2D [29]. Salindri et al. found that well-controlled T2D patients (HbA1c < 8.0%) had faster culture conversion times than those with poorly controlled T2D (HbA1c ≥ 8.0%) in MDR-TB patients [43]. These studies suggest that the adverse effects of T2D on TB disease are attributed at least in part to poor glycemic control and that improving glycemic control may lead to better TB treatment outcomes and a reduced risk of relapse and recurrence. However, achieving optimal clinical management of T2D patients in low- and middle-income countries is challenging, with currently less than 10% of T2D patients receiving guideline-based comprehensive diabetes treatment [44].

Short- versus long-term exposure to hyperglycemia could impact the host immune responses and TB outcomes differently. In vitro, the incubation of mouse bone marrow-derived macrophages (BMDMs) under high glucose conditions (25 mM Glucose) for a short time (48 h) has been shown to reduce TNF-α production, whereas a longer incubation time (7 days) released higher TNF-α compared to BMDMs maintained in baseline 5.5 mM glucose [45]. In a murine model, Martens et al. showed that chronic (≥12 weeks), but not acute (<4 weeks) hyperglycemia, results in a higher bacterial burden and higher inflammation in the lungs compared to normoglycemic controls [46]. Similarly, Cheekatla et al. showed higher lung bacillary load and pathology in hyperglycemic mice compared to control mice at 6 months, but not at 1 and 3 months post-infection [47]. These studies used a streptozotocin-induced model of diabetes. A 12-week high-fat-diet-based murine model of pre-diabetes showed a trend towards a higher Mtb burden in animals with impaired glucose tolerance, significantly higher lung pathology scores and impaired cytokine responses both in the lung and in the blood [39]. Interestingly, the restoration of glucose tolerance while maintaining high body fat conferred resistance to TB in the murine model described above. Immune dysfunction to TB has been confirmed not only in T2D patients, but also in pre-diabetes patients [48].

The question of how hyperglycemia contributes to impaired immune responses to Mtb has been the focus of several studies, with many of them showing functional defects in macrophages, including reduced phagocytosis of Mtb and Mtb killing in diabetic macrophages from both human and animal origins [49,50,51,52]. Interestingly, monocyte-derived macrophages (MDMs) from obese individuals had a higher antigen-presenting capacity to stimulate T cells, whereas those from patients with a “chronic” history of T2D had a compromised capacity for killing intracellular Mtb [53]. Valtierra-Alvarado et al. observed a lower expression of HLA-DR and CD68 on both human monocytes and MDMs from T2D patients. HLA-DR expression in monocytes correlated negatively with HbA1c, VLDL-C and triglyceride concentrations, but HLA-DR and CD68 correlated positively with HDL-C [54]. Restrepo et al. demonstrated reduced HLA-DR expression in diabetic monocytes, even after controlling for BMI and HDL-C. [55]. Altogether, these findings highlight that hyperglycemia cannot be studied in isolation without assessing the impact of dyslipidemia in the susceptibility of T2D patients to TB.

### 4.2. Transient TB-Induced Hyperglycemia

Active TB itself can induce transient stress-hyperglycemia, which usually normalises with TB treatment and does not require long-term diabetes management. Between 17% and 87% of TB patients who have not been previously diagnosed with T2D have elevated blood glucose measurements upon TB diagnosis [56]. However, it is important to follow TB patients longitudinally throughout TB treatment and to determine whether they indeed have newly diagnosed T2D, which requires clinical management, or transient stress hyperglycemia, which resolves with TB treatment. Therefore, only repeated measurements of random or fasting blood glucose and HbA1c throughout TB treatment are confirmative of T2D. The TANDEM study involved such a longitudinal follow-up of TB patients across four different continents and showed that the prevalence of T2D amongst TB patients was lowest in South Africa (10.9%) and highest in Indonesia (19.7%) [57]. In a different study from South Africa, hyperglycemia was transient in the majority of participants with newly diagnosed hyperglycemia, with the median HbA1c found to be significantly decreased at 3 months follow-up (5.7% vs. 5.4%, *p* < 0.0001), whereas patients with pre-existing T2D maintained high levels of blood glucose 3 months after treatment (4.6% vs. 4.7%) [58].

A recent systematic review, revealed that in 50% of TB patients with newly diagnosed hyperglycemia the abnormal glucose tolerance is reversible at 3–6 months’ follow-up with the unresolved total burden of hyperglycemia being at slightly above 10% [59]. These data suggest that a high proportion of TB patients with elevated blood glucose levels at baseline have transient hyperglycemia that can be resolved following effective TB therapy. Stress hyperglycemia even in the absence of T2D is a predictor of mortality during sepsis [60]. Therefore, further studies are urgently needed to comprehensively assess TB treatment outcomes and relapse risks in TB patients with transient hyperglycemia.

Whether TB increases the risk of developing T2D is not conclusively known, although a higher risk of T2D has been suggested in individuals with a history of TB [12,61]. It has been proposed that the stress-induced transient hyperglycemia induces pancreatic β-cell apoptosis, which may predispose those individuals to the development of T2D in the future [62].

## 5. Hyperinsulinemia and TB Disease Severity

Hyperinsulinemia is described as elevated concentrations of circulating insulin in the blood due to insulin resistance and is common in obesity and the early stages of T2D. Hyperinsulinemia goes hand in hand with hyperglycemia and dyslipidemia in T2D; therefore, its independent contribution to TB susceptibility is difficult to assess. In addition, most studies on TB and T2D do not measure fasting insulin levels in patients; therefore, there are few reports in the literature on the association between serum insulin concentrations or insulin resistance and TB disease severity. A recent study among TB patients with T2D patients, stratified according to the degree of insulin resistance and showed that the degree of insulin resistance reflects TB disease severity [63]. Whether elevated insulin concentrations and insulin resistance impact the manifestation of TB in patients without T2D remains to be elucidated. However, high-fat-diet-fed mice with hyperinsulinemia and impaired glucose tolerance had more severe lung pathology compared to control animals even in the absence of full-blown diabetes [64]. Insulin can have both pro- and anti-inflammatory properties and is a known modulator of immune function [65]. Reports of T2D patients developing a TB granuloma at the site of insulin injection suggests that insulin may contribute to TB reactivation [66]. Whether hyperinsulinemia is linked to more severe TB disease and adverse TB treatment outcomes remains to be elucidated.

## 6. Dyslipidemia and TB Disease Severity: Cholesterol vs. Triglycerides

T2D is not only characterised by hyperglycemia and hyperinsulinemia, but also by hyperlipidemia. To complicate matters, the form of dyslipidemia is highly variable in T2D patients across different ethnicities. For instance, TB household contacts with T2D patients in South Africa mainly have elevated cholesterol, whereas diabetic TB contacts in South Texas mainly have elevated triglycerides [7]. The relative contribution of these different forms of dyslipidemia to susceptibility to TB remain to be investigated.

However, there is some evidence suggesting that elevated cholesterol is protective in the context of TB. A large population-based longitudinal study from South Korea, including more than 5 million participants from 2009 to 2018, identified a clear relationship between low total cholesterol levels and a high risk of TB. Interestingly, the correlation was seen to be less robust in T2D and obesity and was lost in subjects receiving statins [67], suggesting that altered lipid profiles in metabolic conditions (obesity, T2D) or with drug treatment (Statin) can affect the susceptibility to TB. In active TB patients, cholesterol concentrations are generally lower compared to healthy controls likely due to TB-associated wasting. Nevertheless, higher cholesterol among TB patients is associated with lower TB disease severity. For instance, the radiological extent of disease was inversely correlated with both HDL and LDL but was not associated with sputum smear grading [68]. Low LDL and HDL were associated with granuloma necrosis and fibroplasia, leading to exacerbated lung damage in TB patients and especially those with T2D [69]. Higher serum cholesterol concentrations, on the other hand, were linked to lower concentrations of serum inflammatory markers and TB-related mortality, and this was independent of BMI [70].

In contrast to the seemingly beneficial effect of cholesterol on TB disease severity, elevated triglycerides appear to be associated with adverse TB treatment outcomes. Higher triglyceride concentrations and lower concentrations of cholesteryl esters were found in TB patients who subsequently failed treatment compared to those who were cured, with two cholesterol esters (16:0 and 18:2) having predictive accuracy of treatment failure at TB diagnosis [71].

The underlying mechanisms addressing the association between high cholesterol levels and reduced TB disease severity are not well elucidated. Cholesterol is essential for the phagocytosis of Mtb by macrophages [72] and elevated total cholesterol can result in the elevation of specific oxidised cholesterols that have been shown to both increase phagocytosis, but also to reduce the growth of Mtb and *Mycobacterium bovis* BCG in human monocytes [73]. Moreover, in vitro supplementation with cholesterol leads to an upregulation of HLA-DR expression in human blood monocytes [74], possibly facilitating antigen presentation. Consistently with this, ex vivo phenotyping of human monocytes revealed that although triglycerides are associated with reduced HLA-DR, cholesterols counterbalance this effect [55]. 

**Figure 1:**
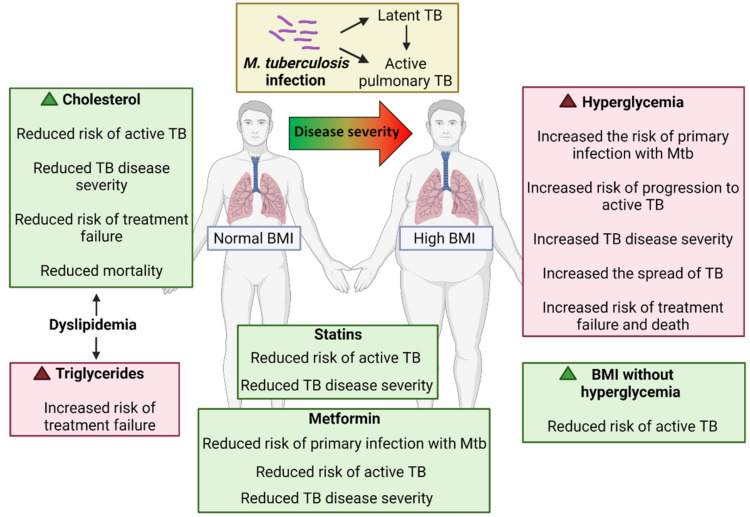
Schematic overview of the impact of cholesterol, triglycerides, hyperglycemia and BMI on risk of active TB, TB disease severity and treatment outcomes. Created with BioRender.com.

Conflicting data have been published about the connection between a cholesterol-rich diet and TB. A randomised control trial showed that adult TB patients receiving a cholesterol-rich diet experienced accelerated sterilisation of sputum Mtb cultures during TB treatment relative to controls receiving a normal diet; however, this trial consisted of a small sample size of 10 and 11 participants per arm [75]. Another population-based study in Singapore reported that a high-cholesterol diet could increase the TB risk [76], in line with a preclinical study showing that hypercholesterolemia impairs immunity to TB in a murine model [77]. Table 1 summarises key studies reporting an association between hyperglycemia and different types of dyslipidemia on TB susceptibility and Figure 1 shows a schematic overview of these findings.

## 7. Impact of T2D Treatment on TB Outcomes

T2D patients are frequently on both glucose-lowering and lipid-lowering medication. Two of the most frequently used T2D treatments, metformin and statins, have been evaluated in relation to TB disease severity and treatment outcomes and have recently received attention as possible candidates for host-directed therapy for TB treatment.

### 7.1. Metformin

Metformin is a glucose-lowering agent that is widely used among patients with T2D. The use of metformin in T2D patients was associated with lower LTBI prevalence. Magee et al. found that using metformin plus two or more other diabetes medications was associated with lower odds of LTBI (adjusted OR 3.9, 95% CI 1.1–13.8), compared to those without any diabetes medication [78].

Metformin use was also associated with a lower risk of developing active TB among TB contacts with T2D [79,80]. T2D patients who included metformin in their treatment strategy had a significantly lower risk of TB compared to those not using metformin. In contrast, insulin users had a significantly higher risk of TB compared to those without insulin use [80]. A recent large study involving over 75,000 T2D patients confirmed the protective effect of metformin; however, only at the highest cumulative dose, whereas lower metformin doses did not reduce the incidence of active TB within a timeframe of two years [81]. In TB patients, metformin use was associated with significantly improved sputum culture conversion rates, fewer pulmonary cavities and reduced mortality rates [31,82,83]. Importantly, these positive effects have been shown to be independent of the degree of blood glucose control [83], suggesting other possible underlying mechanisms mediated by metformin, besides the direct effects on blood glucose control, could underpin the improvement of TB outcomes.

Mechanistically, it has been previously shown that metformin activates adenosine monophosphate-activated protein kinase (AMPK) [84], a regulator of cellular autophagy [85], which is crucial for an effective host innate immune response against intracellular pathogens such as Mtb. Singhal and colleagues showed that metformin inhibits intracellular Mtb growth by inducing ROS and autophagy [82]. Furthermore, Lachmandas et al. observed the strong upregulation of genes involved in phagocytosis and ROS production in PBMC treated ex vivo with metformin [86]. In a murine model, metformin reduced the Mtb burden in the lung (both as a monotherapy and in conjunction with anti-mycobacterials) and improved lung pathology [82].

A beneficial effect of metformin on in vivo Mtb clearance was also shown by Bohme et al. In Mtb-infected mice that received metformin along with pyrazinamide and isoniazid for 30 days, the bacterial burden was compared to mice that had received only pyrazinamide and isoniazid [87], confirming that metformin can enhance the sterilising activity of available antimicrobial treatment for Mtb infection. Conducting subsequent mechanistic experiments, the authors revealed that metformin enhances the host immune function against Mtb by reprograming CD8(+) T cell metabolism, favouring the expansion of the memory CD8+CXCR3+ T cell population with anti-Mtb properties. The increased frequency of this distinct memory T cell phenotype has been consistently observed in both metformin-treated mice, as well as in PBMC from metformin-treated T2D patients [87].

A question remains as to whether the beneficial effects of metformin can only be achieved in TB patients with T2D or whether metformin can also improve TB outcomes in non-diabetic subjects. There are no data yet from ongoing human clinical trials. Preclinical studies, however, show conflicting results. Although Singhal et al. reported a reduction in the lung bacillary load in euglycemic mice receiving metformin either alone or in combination with TB drugs [82], a recent study reported that metformin improves TB severity only in hyperglycemic mice and not in non-diabetic control animals [88]. The authors showed that the treatment of diabetic mice with metformin reduced the Mtb in the lung burden by ~1.5log CFUs compared with untreated hyperglycemic mice, but strikingly augmented lung bacterial loads and immunopathology in nondiabetic mice [88]. Another study provided evidence that metformin has no significant effect on mice receiving the first-line TB regimen [89]. Taken together, these results further consolidate the evidence that hyperglycemia itself increases TB severity and suggest that metformin may be beneficial for improving TB severity and treatment outcomes, at least in patients with TB and T2D comorbidity. Data from clinical trials assessing the utility of metformin as an adjunct TB treatment in non-diabetic patients are urgently needed to conclusively confirm or disregard metformin as a host-directed TB therapy.

### 7.2. Statins

Statins lower cholesterol levels by inhibiting the 3-hydroxy-3-methylglutaryl-CoA reductase, one of the key enzymes in the generation of cholesterol, but also have anti-inflammatory properties. Statins are one of the most frequently prescribed drugs to reduce morbidity and mortality in patients with hypercholesterolemia, coronary heart disease, T2D patients and in patients suffering from infectious diseases [90,91,92].

Statin therapy significantly reduced the risk of TB in T2D patients by 22% (pooled RR 0.78, 95% CI 0.63–0.95) and TB patients without T2D by 40% (pooled RR 0.60, 95% CI 0.50–0.71) [93]. Pan et al. demonstrated that statin use was associated with a 35% decreased risk of TB (crude HR, 0.648; 95% CI, 0.430–0.976) compared with no statin use as the reference group [94]. Macrophages isolated from patients with hypercholesterolemia receiving daily statin therapy and infected with Mtb in vitro had significantly lower Mtb growth 3 days post-infection compared to the controls [95]. Statin therapy in mice led to a reduction in the Mtb burden in the lung, liver and spleen at 4 weeks and 8 weeks post-infection compared with mice that received the vehicle control [95]. This was further confirmed by Skerry et al., who demonstrated that the treatment of Mtb-infected macrophages with simvastatin significantly reduced the bacterial load compared to the vehicle, and this was further enhanced by the bactericidal activity of isoniazid [96]. In chronically infected mice (6 weeks post-infection), a standard oral treatment regimen of rifampicin, isoniazid and pyrazinamide along with simvastatin demonstrated greater bacillary killing in the lungs, compared to treatment without simvastatin at 4 weeks (*p* < 0.01) and 8 weeks (*p* < 0.01) of treatment [96]. However, in a retrospective study in South Korea, there was no evidence that the use of statins provided a protective effect on TB incidence (aHR 0.98; 95%CI 0.89–1.07). Differences in these results may be due to the ethnicities of participants, baseline metabolic characteristics of the participants and trial interventions [97].

Combined metformin and statin use in patients with diabetes was associated with less than half the prevalence of LTBI (4% combined metformin/statin) compared to no treatment (10%) [78]. There may be benefits for patients with T2D at risk of LTBI using a combination therapy of both metformin and statins, as preventing LTBI is an essential step for preventing active TB disease and both LTBI and TB are complicated by T2Ds [78]. 

### 7.3. Aspirin

T2D is a known risk factor for cardiovascular events and studies have shown that TB itself is also associated with an increased risk of ischemic stroke and myocardial infarction [98,99,100,101], even after a successful TB treatment course [100]. TB-T2D co-morbidity is expected to significantly increase the risk of cardiovascular diseases; however, cardiovascular risk and appropriate therapeutic interventions are poorly studied in patients with TB-T2D [102].

Aspirin remains widely prescribed for the prevention of cardiovascular and cerebrovascular events in T2D patients and has also been proposed as an adjunctive therapy to current anti-TB treatments. The administration of low-dose aspirin in C3HeB/FeJ mice infected with Mtb both alone or together with anti-TB treatment increased the survival rate [101]. In addition, animals receiving aspirin had decreased lung bacterial burdens and pathology at 3–4 weeks after infection, accompanied by reduced systemic inflammatory cytokines (IL-6, IL-1β and TNF-α) and increased Arg-1/INOS staining on lung sections, which is consistent with the anti-inflammatory effect of aspirin. Marzo et al. reported that aspirin administered with ibuprofen reduced the bacterial load, lung pathology, concentrations of pro-inflammatory cytokines and improved survival in Mtb-infected mice [103]. 

In TB patients the use of anti-platelet aggregating drugs, including aspirin, was linked to lower rates of smear positivity, fewer cavities and reduced 12-month mortality rates [104]. The only randomised controlled trial in pulmonary TB patients with T2D showed that aspirin could improve the clinical efficacy of standard anti-TB treatment by increasing the rate of sputum conversion, significantly reducing the number of cases with cavities, as well as the number of cavities during the first 2 months of therapy [98,99,105].

## 8. Conclusions

There are conclusive data for an increased risk of T2D patients to primary infection with Mtb, to the progression from LTBI to active TB and to adverse TB treatment outcomes. Although hyperglycemia was previously thought to be the main driver of increased susceptibility to TB, there is now mounting evidence that other host metabolic factors such as hypercholesterinemia and elevated triglycerides further modify this risk of increased susceptibility to TB in T2D patients. Although high triglycerides (similar to hyperglycemia) are associated with adverse TB treatment outcomes, high cholesterol is suggested to have a favourable effect on the clinical manifestation and outcomes of TB treatment. Further studies are needed to fully dissect the relative contributions of the metabolic drivers in immune responses to Mtb. Hyperglycemia and various forms of dyslipidemia frequently occur concurrently in humans, making it challenging to dissect the relative contributions of these metabolic factors to susceptibility to TB. Therefore, the development of novel animal models that allow the dissection of these metabolic factors in the context of Mtb infection are urgently needed.

## Figures and Tables

**Table 1:** Impact of hyperglycemia and different types of dyslipidemia on TB susceptibility.

Host	Hyperglycemia	Impact on TB Susceptibility	Refs.
Human	T2D patient with high HbA1c (≥9%)	Elevated risk of infection and hospitalisation, increased mortality, lower rate of sputum culture conversion	[29,41,42]
	T2D patients with controlled HbA1c (≤8%)	Reduced risk of hospitalisation, faster sputum culture conversion time	[41,43]
	MDMs from obese humans	Higher antigen-presenting capacity to stimulate T cells	[53]
	Monocytes, MDMs from T2D patients	Compromised capacity for killing intracellular Mtb, lower expression of HLA-DR and CD68, HLA-DR expression correlated negatively with HbA1c, VLDL-C and triglyceride concentrations, but HLA-DR and CD68 correlated positively with HDL-C	[53,54,55]
Mouse	Chronic hyperglycemia (≥12 weeks); STZ model	Higher bacterial burden and higher inflammation in the lungs compared to acute hyperglycemia (STZ treatment for 4–9 weeks)	[46,47]
	Pre-diabetes (≤8% HbA1c); impaired glucose tolerance; HFD model	Trend towards higher Mtb burden in animals with impaired glucose tolerance, significantly higher lung pathology scores and impaired cytokine responses	[39]
**Host**	**Dyslipidemia**	**Impact on TB susceptibility**	**Refs.**
Human	Low TC	Associated with an increased risk of TB disease	[67]
	Low TC, HDL-C, LDL-C	Associated with more extensive lung lesions on chest radiographs/CT scans, higher degree of smear positivity	[68,69]
	High TC, HDL-C, LDL-C	Associated with lower all-cause and infection-related mortality, reduced levels of inflammation markers	[70]
Mouse	High TC	Associated with delayed expression of adaptive immunity	[77]

TC, total cholesterol; VLDL-C, very-low-density lipoprotein cholesterol; LDL, low-density lipoprotein cholesterol; HDL, high-density lipoprotein cholesterol; STZ, streptozotocin; HFD, high fat diet.

## Data Availability

Not applicable.
